# Opening editorial

**DOI:** 10.1186/s12967-017-1126-7

**Published:** 2017-02-01

**Authors:** Seah H. Lim

**Affiliations:** Division of Hematology and Oncology, Rhode Island Hospital/Brown University Warren Alpert Medical School, Providence, RI 02903 USA

I am excited to announce the launch of *Translational Hematology*, a new section of the *Journal of Translational Medicine*. This section aims to support translational research in the discipline of Hematology. It will encompass research in the fields of Red Blood Cells, White Blood Cells, Thrombosis and Hemostasis, Transfusion Medicine, and Hematopoietic Progenitor Cell Transplantation.

Hematology is the study of normal hematopoiesis, and the pathology and diseases of the blood, lymphatic system, and bone marrow. It has always been a discipline that has incessantly promoted translational research, especially in the last two decades. Studies in red blood cells have progressed from the simple microscopic examination of morphology and physical evaluation of rheology to the genetic therapy of inherited red blood cell disorders and modern iron chelating agents. Therapy for various hematologic malignancies has also evolved from the use of non-specific chemotherapeutic agents to selective molecular and immune targeting. The availability of new recombinant coagulation factors for congenital bleeding disorders, novel oral anticoagulants for thrombotic disorders, and artificial blood products instead of homologous blood will all provide the opportunity for interesting and clinically relevant translational research that will change clinical practice. Selective T cell depletion to prevent graft-versus-host disease without compromising the graft-versus-tumor effects, ex vivo graft engineering and hematopoietic progenitor cell expansion, and the safer and wider applicability of haplo-identical hematopoietic progenitor cell transplant are just a few of the many active translational research activities ongoing. The recent discovery of the effects of the intestinal microbiota on both sickle cell vaso-occlusive crisis and the development of graft-versus-host disease following allogeneic hematopoietic progenitor cell transplant further supports the interfacing of hematology with other disciplines. The vast range of translational research in hematology, therefore, provides the basis for the establishment of a specially dedicated forum such as *Translational Hematology* to not only bring together investigators in translational hematology but also to provide the framework for interaction between investigators from multi-disciplines within one single journal.

The *Translational Hematology* section of the *Journal of Translational Medicine* aims to provide the vehicle to disseminate research data on benign and malignant hematology, and transfusion medicine. Since hematology is a very diverse discipline that includes cellular biology, molecular biology, and transplantation immunology, we expect that the *Translational Hematology* section will be a very dynamic section of the *Journal of Translational Medicine*. The section should, therefore, be of great interest to investigators in both hematology and many other disciplines. We are particularly interested in promoting research results from novel pre-clinical and early clinical translational efforts. New ideas, hypothesis-driven perspectives, and reviews on any particular aspects of hematology are also highly desirable and encouraged because we believe that such contributions will provide the platform for further discussions and hypothesis-testing to hasten our efforts to seamlessly bring successes from bench to bedside.

The mission of the *Journal of Translational Medicine* to provide rapid publications, open-access, and high standard peer-review process should provide an ideal platform for a highly interactive and timely discussion among investigators. The *Translational Hematology* section is proud that its Editorial Board is made up of many high caliber basic and physician scientists of top class scientific and translational reputation in their respective areas of hematology. This will definitely ensure that every published manuscript is of the highest quality and internationally competitive. On behalf of the Editorial Board, I look forward to receiving your contributions to this forum.

Seah H Lim MD PhD FRCP (Lond.) FRCPath. FACP, Section Editor, *Translation Hematology*.

